# Shifting Paradigms in the Mechanics of Nectar Extraction and Hummingbird Bill Morphology

**DOI:** 10.1093/iob/oby006

**Published:** 2019-01-02

**Authors:** A Rico-Guevara, M A Rubega, K J Hurme, R Dudley

**Affiliations:** 1 Department of Integrative Biology, University of California, Berkeley, 3040 Valley Life Sciences Building, Berkeley, CA 94720, USA; 2 Department of Ecology and Evolutionary Biology, University of Connecticut, Storrs, CT 06269, USA; 3 Instituto de Ciencias Naturales, Universidad Nacional de Colombia, Código Postal 11001, Bogotá DC, Colombia

## Abstract

As functional morphologists, we aim to connect structures, mechanisms, and emergent higher-scale phenomena (e.g., behavior), with the ulterior motive of addressing evolutionary patterns. The fit between flowers and hummingbird bills has long been used as an example of impressive co-evolution, and hence hummingbirds’ foraging behavior and ecological associations have been the subject of intense study. To date, models of hummingbird foraging have been based on the almost two-centuries-old assumption that capillary rise loads nectar into hummingbird tongue grooves. Furthermore, the role of the bill in the drinking process has been overlooked, instead considering it as the mere vehicle with which to traverse the corolla and access the nectar chamber. As a scientific community, we have been making incorrect assumptions about the basic aspects of how hummingbirds extract nectar from flowers. In this article, we summarize recent advances on drinking biomechanics, morphological and ecological patterns, and selective forces involved in the shaping of the hummingbird feeding apparatus, and also address its modifications in a previously unexpected context, namely conspecific and heterospecific fighting. We explore questions such as: how do the mechanics of feeding define the limits and adaptive consequences of foraging behaviors? Which are the selective forces that drive bill and tongue shape, and associated sexually dimorphic traits? And finally, what are the proximate and ultimate causes of their foraging strategies, including exploitative and interference competition? Increasing our knowledge of morphology, mechanics, and diversity of hummingbird feeding structures will have implications for understanding the ecology and evolution of these remarkable animals.

## Introduction

Hummingbirds are increasingly exciting subjects with which to study interactions among morphology, behavior, and ecology. Compared to other vertebrates, they present researchers with examples of extreme morphological design (being some of the smallest endotherms), physiological limits (e.g., the highest vertebrate mass-specific metabolic rates while hovering, Lasiewski 1963; Suarez 1992; the highest vertebrate sucrase activity, Nicolson and Fleming 2014; the smallest amniote genomes, Gregory et al. 2009; Wright et al. 2014), and locomotor performance (e.g., hovering flight and but also high levels of maneuverability, Weis-Fogh 1972; Chai and Dudley 1999; Tobalske et al. 2003; Hedrick et al. 2009; Dakin et al. 2018). Variation in hummingbird bill morphology is similarly impressive, with almost 20-fold range in length (e.g., comparison of Purple-backed Thornbill [*Ramphomicron microrhynchum*, ∼6 mm] to the Sword-billed Hummingbird [*Ensifera ensifera*, ∼110 mm]), with bill curvature varying from being only slightly upturned at the tip to a sweeping 90° decurved angle (e.g., comparison of the Fiery-tailed Awlbill [*Avocettula recurvirostris*] to the White-tipped Sicklebill [*Eutoxeres aquila*]), and even the presence of hooks, daggers, and serrations on male bill tips in some species (e.g., Tooth-billed Hummingbird [*Androdon aequatorialis*] and Saw-billed Hermit [*Ramphodon naevius*], Rico-Guevara and Rubega 2017).

Hummingbirds have long been considered as classic textbook examples of coevolution between flowers and their pollinators, given the close match between flowers and bill shapes (e.g., Futuyma 2009). Scientists have studied nectar foraging in hummingbirds for several decades; and these studies have increased our understanding in several respects, such as nectar characteristics and preferences (e.g., Stiles 1976; Hainsworth and Wolf 1976; Calder 1979; Tamm and Gass 1986; Martínez del Río 1990; Stromberg and Johnsen 1990; Roberts 1996; Blem et al. 1997; Blem et al. 2000; Fleming et al. 2004), foraging optimization (e.g., DeBenedictis et al. 1978; Gass and Roberts 1992; Hixon and Carpenter 1988; Baum and Grant 2001; Bateson et al. 2003), and extraction efficiency (e.g., Ewald and Williams 1982; Roberts 1995). Long-held paradigms about the underpinnings of hummingbird feeding mechanics relative to their energetics and feeding ecology have recently been transformed (Rico-Guevara and Rubega 2011, 2012; Rico-Guevara et al. 2015). Altering our paradigms about nectar intake mechanics, and selective pressures on feeding structures (e.g., bill and tongue), must necessarily influence core assumptions regarding efficiency, optimality, and ultimately coevolution.

In general terms, it has been shown that the main evolutionary driver of bill morphology in birds is feeding ecology (reviews in Cooney et al. 2017; Olsen 2017). Although the feeding ecology of hummingbirds may not seem as diverse as in other groups of birds such as waterfowl, or finches, which feed on a variety of food items (see Bowman 1961; Olsen 2017), hummingbird beak shape variation (as described above) is remarkable. We propose here that diversity of beak morphology in hummingbirds is not only linked to the bill/corolla match, but also is influenced by their foraging strategies. Several behavioral tactics employed by hummingbirds foraging for nectar have been identified (e.g., Feinsinger and Colwell 1978; Lanna et al. 2017). These foraging strategies ultimately differ in the kind of competition (or their relative proportions) employed by a given individual; hummingbirds engage in both exploitative and interference competition. Optimal foraging at the floral visitation level predicts a specific set of changes in beak shape that will elevate the rate of energy gain, but bird beaks are however used in a variety of contexts (see Olsen 2017). Of special relevance here, some birds use their bills as weapons (see Rico-Guevara and Hurme 2018). So, there is an additional selective pressure on the evolution of hummingbird bill shape that comes from fighting with their beaks (e.g., Rico-Guevara and Araya-Salas 2015; Hurme and Rico-Guevara 2016; Araya-Salas et al. 2018). We propose exploitative and interference competition to be competing hypotheses for the evolution of particular traits in hummingbird bills. In this article, we delineate potential trade-offs in the evolution of bill shapes adapted for optimal nectar extraction vs. intrasexual fighting and interference competition (e.g., Rico-Guevara and Hurme 2018). We currently have the technological (e.g., Rico‐Guevara and Mickley 2017) and conceptual tools to continue advancing our understanding of these various factors in the evolution of hummingbird-plant systems. Since hummingbird morphology primarily evolved to enable rapid floral visitation, it is of fundamental importance to understand mechanistically the function and mechanical performance of nectar-feeding and its implications. In addition, since floral visitation is inextricably linked to hummingbird competitive behaviors both through exploitation and interference, a holistic assessment of their morphological and behavioral adaptations to this end is necessary.

## Recent advances in our understanding of nectar collecting mechanisms

**Fig. 1:**
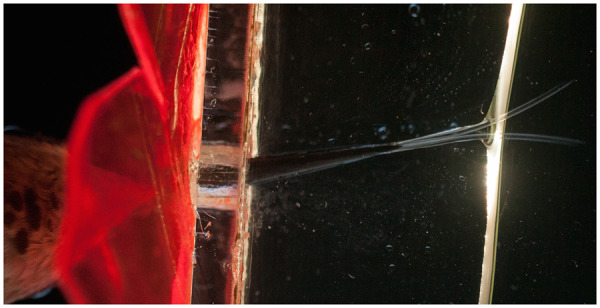
Hummingbird feeding apparatus. Ventral photograph of the bill and tongue of a female Anna’s hummingbird (*Calypte anna*), courtesy of Don Carroll. This picture was taken of a bird at a feeder from below; the bird’s throat is at the lower left, and the bifurcated tongue, immersed in nectar, is in the upper right. Note that the tongue is composed of two identical grooves, and that the lingual tissue is transparent. Interestingly, the portions of the hummingbird feeding apparatus that fit inside the flower have no intrinsic muscular control (Rico-Guevara 2017), and are instead controlled remotely by the hyoid apparatus muscles (tongue reciprocation) and by the cranial muscles involved in bill kinesis (opening and closing). Additional kinetic events at the distal portions of the tongue (e.g., tongue tip separation when in contact with the nectar) derive from biophysical interactions.

**Fig. 2:**
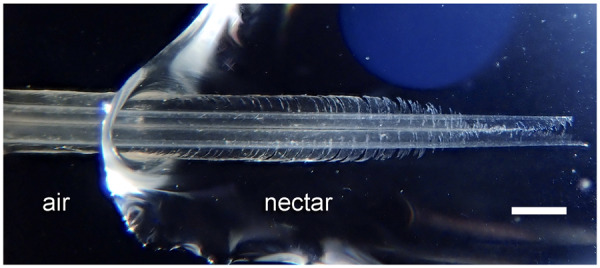
Tongue tip morphology. Photograph of the tongue of a male Ruby-throated Hummingbird (*Archilochus colubris*); scale bar = 0.5 mm. Thin, transparent, and curled tissue forms the tongue grooves (see Rico-Guevara 2017). Note that one edge of each groove is fringed near the tip. This photo was taken on a 1 mm deep nectar film to exemplify how the tongue tip would spread on a thin nectar layer found inside a flower.

Since hummingbirds collect floral nectar using their tongues, we expect that this organ will correspondingly reflect mechanical specialization for fluid uptake. Historically, it has long been recognized that hummingbird tongues are long, thin, and distally forked (Fig. 1; Martin 1833; Darwin 1841). The distal one-third to one-half of the tongue is divided into two longitudinal and parallel open-sided grooves, each of which terminates distally in a fringed region (Lucas 1891; Fig. 2). The distal grooves do not reach posteriorly into the tongue base (Scharnke 1931; Weymouth et al. 1964), and hummingbirds therefore cannot employ differential air pressure to move liquid through their tongues and subsequently into the pharynx (i.e., the tongue grooves do not function as drinking straws). Prior authors have suggested nectar could flow through these tiny tubes via capillary action (Scharnke 1931; Weymouth et al. 1964; Hainsworth 1973; Ewald and Williams 1982). In a comprehensive model for nectar intake, Kingsolver and Daniel (1983) formalized the idea that capillarity is the mechanism by which the nectar passes from the floral nectar chamber to the birds’ tongue. This view of nectar feeding mechanics in hummingbirds has been widely accepted but was never tested empirically until recently.

Our studies on nectar feeding in freely feeding hummingbirds established that capillarity cannot explain the movement of nectar onto the tongue tips and into the tongue grooves (Rico-Guevara and Rubega 2011, 2012). Instead, the functional traits that make hummingbird tongues highly efficient at extracting liquid derive from the material properties and structural configuration of the tongue tip and grooves (Rico-Guevara and Rubega 2011; Vogel 2011; Rico-Guevara et al. 2015; Rico-Guevara 2017). Other aspects of the feeding process await further explanation (see Rico-Guevara and Rubega 2017), and patterns of mechanistic variation across hummingbird species (with consequent ecological implications) remain to be described. Here, we summarize these recent advances in understanding the nectar intake process in hummingbirds.

### Fluid trapping

Initially in these studies, hummingbird tongues were shown to trap nectar at the tongue tips (Rico-Guevara and Rubega 2011). The authors filmed free-living birds with high-speed video (at speeds up to 2400 frames/s) to document tongues moving in and out of nectar (hereafter, in vivo observations). The authors also used tongues removed from salvaged carcasses of dead hummingbirds (hereafter, post mortem observations) to emulate movements of the tongue at the air–nectar interface under controlled laboratory conditions. Both in vivo and post mortem videos revealed that upon contact with fluid, the tongue fringes immediately unfurl, the tips separate, and the whole tongue tip spreads outwards, to cover a large area, even in thin nectar films (Fig. 2). As the tongue is then withdrawn from the fluid, surface tension causes each fringe to initiate closing just before passing the air-nectar interface, and to be fully closed at the plane of the interface. Hence, all the fringes have rolled inward by the time withdrawal is complete, thus trapping nectar under each fringe at the tongue tip.

Rico-Guevara and Rubega (2011) noted that the progressively smaller fringing toward the tongue tip imparts a distally closed and conical shape to the furled tip when the tongue is withdrawn from the nectar. The authors surmised that this effect creates a “lingual seal” preventing fluid from dripping out of the tongue, which is a nontrivial consideration given that inertial forces at high licking rates would tend to dislodge fluid from the tongue. This demonstration of dynamic tongue tip mechanics directly contradicts the capillarity hypothesis, which mandates more static “tongue tubes” (Martin 1833; Scharnke 1931; Weymouth et al. 1964; Hainsworth 1973; Ewald and Williams 1982; Kingsolver and Daniel 1983). The challenge to the standard capillarity paradigm was controversial (as summarized by Nicolson and Fleming 2014). Some researchers, based on scant video evidence drawn from hummingbirds feeding in highly unnatural feeding conditions, continued to argue for the capillarity hypothesis (Kim et al. 2011, 2012), and thus motivated further physical analysis of the nectar collection mechanism in wild hummingbirds (e.g., Rico-Guevara and Rubega 2012, Rico-Guevara et al. 2015).

### Elastic pumping

**Fig. 3:**
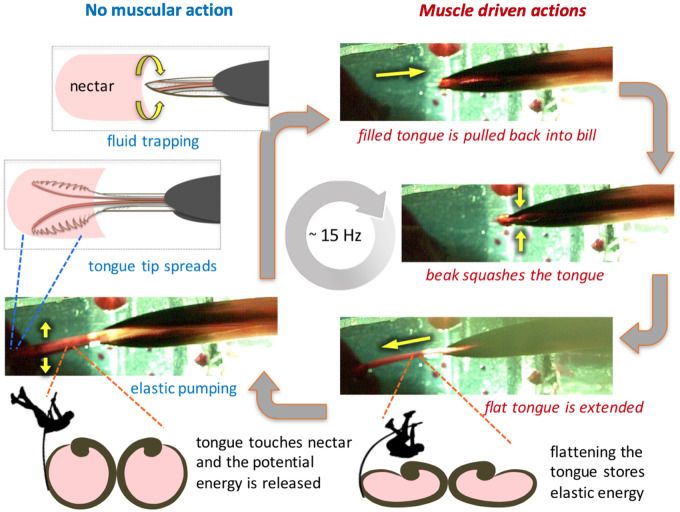
Licking cycle in hummingbirds. On the left and in blue font, the figure portrays those biophysical processes that fill the tongue with nectar (i.e., fluid trapping, Rico-Guevara and Rubega 2011; elastic pumping, Rico-Guevara et al. 2015). These fluid motions require no extra energy expenditure (i.e., no muscle participation); they instead derive passively from consequences of the tongue morphological design (i.e., geometrical shape and tissue composition). The muscular actions that reciprocate the tongue, along with the squeezing of the bill tips, are shown on the right and in red font. The tongue cross sections at the bottom with the pole vault cartoons depict how potential energy storage in the tongue via dorsoventral compression is translated into kinetic energy that reshapes the tongue, thus pumping the liquid proximally. Video stills are from a male or female Amazilia Hummingbird (*Amazilia amazilia*) drinking artificial nectar at a licking rate of approximately 15 times a second.

To definitively eliminate capillarity as a biologically relevant mechanism for nectar collection, Rico-Guevara et al. (2015) worked with wild birds under conditions designed to realistically simulate the conditions hummingbirds encounter at flowers, and described how hummingbird tongue grooves function as elastic micropumps and not as capillary tubes. During tongue extrusion, a hummingbird collapses its tongue by squeezing it through its mostly closed bill tips. Measurements on living birds, under conditions that realistically simulated floral morphologies, showed that the tongue grooves remained collapsed until the tongue tips contacted the nectar surface (Rico-Guevara et al. 2015; Fig. 3). After contacting the surface, the grooves expanded and filled completely with nectar; formation of a meniscus within the tongue grooves was not observed (Rico-Guevara et al. 2015). This result is contrary to expectations for capillary filling, which requires the empty and cylindrical tongue grooves contact the nectar surface so that a meniscus is formed. Furthermore, rather than a decrease in groove diameter upon contact with nectar (as predicted by the capillarity hypothesis), groove diameter actually increased significantly (Rico-Guevara 2014; Rico-Guevara et al. 2015). All observed licks followed the same pattern: groove diameter (*i.e.*, tongue thickness) remained constant during protrusion of the tongue, and then rapidly increased after the tips contacted nectar (Fig. 3). The tongue remained compressed prior to nectar transport, and thus no empty space within the tongue was available to fill via capillarity. The reduction in diameter (curling and/or flattening of the semi-cylindrical structure) of the tongue grooves, and consequently of the lumen of each one, is executed in an area of compression near the bill tip (Rico-Guevara and Rubega 2017). This collapsed structural configuration is retained when the tongue is traveling through air, which allows precise quantification of the filling time. Such a compressed state of the grooves is maintained both by the adhesive properties of the liquid layer trapped inside the flattened structure. and by the compression of the basal portion of the grooves still being executed by the bill tips (Rico-Guevara 2014; Rico-Guevara et al. 2015). This compression remains until the tongue tip contacts the nectar surface, when the grooves reshape into their semi-cylindrical configuration by filling their lumina with nectar during their expansion. To explain these physical phenomena, the authors developed an elasto-hydrodynamic model for expansive nectar filling (Rico-Guevara et al. 2015). In particular, the bird loads elastic energy into the groove walls while squeezing nectar off the tongue during protrusion (see Rico-Guevara and Rubega 2017); this energy is subsequently used to pump nectar into the grooves (see Fig. 3).

## Implications for ecological patterns and coevolutionary processes

Because capillarity models do not explain observed features of nectar intake in hummingbird tongues (Rico-Guevara and Rubega 2011; Rico-Guevara et al. 2015), the associated equations that have been used for decades (e.g., Heyneman 1983; Kingsolver and Daniel 1983; Kim et al. 2011, 2012) to calculate rates of energy intake while consuming nectar (as well as estimates of optimal floral nectar concentration in hummingbird-pollinated plants; Nicolson and Fleming 2003) are neither accurate nor appropriate. Given that energy content of nectar increases with sugar concentration, but that flow rate concurrently decreases due to elevated dynamic viscosity, “capillary feeders” would optimize rates of energy intake at intermediate nectar concentrations (Baker 1975; Heyneman 1983; Kingsolver and Daniel 1983; Nicolson and Thornburg 2007; Kim et al. 2011, 2012). In particular, capillarity models for optimal concentrations during nectar extraction by hummingbirds yield values of 20–40% (mass/mass; see Heyneman 1983; Kingsolver and Daniel 1983; Kim et al. 2011). However, hummingbirds given a choice of nectar concentrations in behavioral experiments preferred values of 45–65% (e.g., Van Riper 1958; Pyke and Waser 1981; Tamm and Gass 1986; Roberts 1996; Blem et al. 1997; Blem et al. 2000). In addition, given the strong influence of gravity on tongue filling via capillary action, nectar intake rates should be higher at pendulous flowers, although experiments have failed to demonstrate consistent trends with flower angle (e.g., pendulous, horizontal, and erect flowers; Montgomerie 1984; Collins 2008). Handling times (Montgomerie 1984) and nectar extraction rates (Collins 2008) also do not significantly differ in response to different corolla orientations.

In contrast, both nectar trapping and elastic micropump data (Rico-Guevara et al. 2015) suggest a much weaker trade-off between nectar energy content and uptake rates than that predicted by capillarity equations. These findings instead suggest that hummingbirds should select the highest nectar concentration available to maximize energy intake. Furthermore, the tongue when functioning as an elastic micropump fills an order of magnitude faster than would be expected under capillarity (Rico-Guevara and Rubega 2012; Rico-Guevara et al. 2015), and thus allows for higher energy intake via elevated licking rates and complete tongue filling. The absence of a strong gravitational effect for the tongue-filling mechanism is also better explained by the fluid trapping and elastic pumping models (see Rico-Guevara and Rubega 2011; Rico-Guevara et al. 2015). Furthermore, fluid collection by the tongue is only the first step in a chain of intricate processes that have to work in concert to achieve actual nectar feeding (Rico-Guevara 2014). For instance, liquid offloading occurs upon subsequent tongue protrusion at every lick cycle (Rico-Guevara and Rubega 2017), and then each nectar load has to be moved to where it can be swallowed (intraoral transport). Taken together, these insights suggest that nectar concentration in hummingbird-pollinated flowers will be a result of biophysical constraints not just on the nectar loading of the tongue, but also in subsequent stages of the drinking process by hummingbirds (e.g., intraoral transport), as well as by their taste perception (e.g., Baldwin et al. 2014), and on selective pressures on hummingbird-pollinated plants to balance the cost of providing highly concentrated nectar relative to the likelihood of securing pollination services (see Nicolson 2007).

This new interpretation of the relative costs and benefits of the flower-hummingbird interaction suggests, in turn, important coevolutionary consequences. It is well-known that species coexistence in nature relies on differential use of resources, and this leads to ecological specialization (Futuyma and Moreno 1988; Forister et al. 2012). Morphology of the feeding apparatus thus varies with foraging strategy, as is most evident among dietary specialists which have evolved remarkable adaptations for feeding. Hummingbirds are among the most committed nectar specialists in nature; many extreme aspects of their biology (e.g., hovering capacity, body miniaturization, up-regulated metabolic physiology) revolve around feeding on flowers, and their bills have transformed into elongated cylinders that diverge dramatically in form from those of their relatives. In parallel, many clades of plants have independently evolved long, tubular, brightly colored, and scentless flowers (Stiles 1981; Stiles and Freeman 1993; Nicolson 2002). Just as different plant species may attract the same pollinator, a single plant species may also be pollinated by different hummingbird species, an outcome described as “diffuse coevolution” (Janzen 1980; Lunau 2004).

We highlight the importance of understanding the biomechanics of the hummingbird-flower interaction from both the animal (nectar extraction) and plant (pollination) perspectives. Hummingbirds have clearly influenced macroevolutionary trends in the plants that they visit for nectar (e.g., Tripp and McDade 2013). For instance, some hummingbird-pollinated plant clades show diversification rates higher than those of insect-pollinated plants (Serrano-Serrano et al. 2017). Diffuse coevolution between hummingbirds and plants highlights the importance of understanding the shifting form and functional trends between bills and floral corollas (e.g., configuration of floral petals), which will influence outcomes of the pollinating shape interactions. In addition, intra- and interspecific aggression among pollinators are among the main factors accounting for as much as twice the level of paternal diversity (*i.e.*, multiple paternity) in bird-pollinated vs. insect-pollinated plants (Krauss et al. 2017). There is extensive variation not only in the strength of the association between bill morphology and hummingbird-pollinated plant flowers, but also in the matching of divergence times of both bird and plant clades (e.g., Tripp and McDade 2013; Abrahamczyk et al. 2014; Abrahamczyk and Renner 2015), which highlights the importance of understanding the different selective pressures and evolutionary history of ecological interactions in both plants and hummingbirds. Various floral traits must interact synergistically to influence pollination outcomes. Typically, floral pollinators are viewed along a continuum from generalist to specialist, which for hummingbirds will influence trends in bill evolution. Contrariwise, plants with a generalist pollination strategy will have a wide range of opportunistic or even unfaithful pollinators, whereas plants with a specialist pollination strategy restrict nectar access to only a few specialized pollinators, thereby creating a close coevolutionary association (Proctor et al. 1996; Abrahamczyk et al. 2014). Hummingbird specialist plants (*i.e.*, those possessing trochilophilous flowers), for instance, restrict access via a corolla that well matches both length and shape of hummingbird bills (e.g., Wolf et al. 1972, 1975). Hummingbird and plant species with complementary morphological traits for feeding (*i.e.*, beaks) and reproduction (*i.e.*, floral morphology), respectively, exhibit greater interaction frequencies relative to bird and plant species without bill-corolla matching traits (e.g., Maglianesi et al. 2014), even when other nutritional resources are abundant (e.g., Weinstein and Graham 2017).

Trait matching between bills and corollas circumscribes pollinator-plant coevolution by determining both the frequency and details of ecological interactions among hummingbird and plant species (Wolf et al. 1972, 1975; Stiles 1981). Regardless of the plant’s pollination strategy (*i.e.*, generalist vs. specialist), increased trait-matching may also benefit the plant if there are sites on the hummingbird body to which pollen readily adheres and cannot be easily removed during the trip from flower to flower (e.g., the forehead), and to which anthers and stigma better conform in shape and angle. By varying floral shape and nectar reward, as well as floral spatial distribution (e.g., within an individual plant), plants can influence foraging efficiency of visitors and perhaps even select among potential pollinators to enhance pollen transfer. However, our understanding of the details of hummingbird foraging optimization relative to plant-pollinator coevolutionary patterns and processes remains incomplete. For example, one commonly proposed proximate explanation for trait-matching trends in hummingbirds and flowering plants is that plants with floral traits closely matching corresponding bill morphologies will reward hummingbirds via increased efficiency of nectar extraction (e.g., Maglianesi et al. 2014, Weinstein and Graham 2017). Foraging optimization, defined here as an increase in the gross rate of energy gain and/or a reduction of the rate of energy expenditure in obtaining a given resource (independent of the currency, see Houston and McNamara 2014), would be expected to increase under this proposed regime through coevolutionary processes (e.g., Royama 1970; Tullock 1971; Wolf and Hainsworth 1991; Collins 2008). The rationale behind this bill-corolla trait matching explanation is that individuals of a given species (or sex, intraspecifically) should be more efficient feeders (*i.e.*, have a greater nectar intake rate) when visiting flowers that better match their bill length and shape (e.g., Wolf et al. 1972; Lara and Ornelas 2001; Temeles et al. 2010). However, tests of this bill-corolla matching hypothesis have produced conflicting results (see below).

As an example let us consider the match between bill and corolla lengths. One might assume that long-billed and short-billed hummingbird species would feed more efficiently on long and short flowers, respectively, under this hypothesis. However, this prediction has never gained full empirical support (e.g., Hainsworth 1973; Montgomerie 1984; Temeles and Roberts 1993; Temeles 1996), meaning that longer-billed birds do feed more quickly from longer ﬂowers relative to shorter-billed birds, but also that shorter-billed birds do not more rapidly feed from shorter ﬂowers when compared to longer-billed birds (see Temeles 1996; Temeles et al. 2009). Biomechanically, birds with longer bills could achieve shorter distances between the bill tip and nectar rewards when probing inside deep corollas (Wolf et al. 1972; Montgomerie 1984). Such reduced distances for tongue displacement yield greater licking rates (Ewald and Williams 1982; Collins 2008) and enhanced nectar extraction efficiency (Hainsworth 1973; Hainsworth and Wolf 1976; Montgomerie 1984; Grant and Temeles 1992; Temeles and Roberts 1993; Temeles 1996). Furthermore, the elastic micropump mechanism that fills with nectar those portions of the tongue grooves remaining outside the fluid (Rico-Guevara et al. 2015) must be optimized to work at relatively short distances. When the bill tip-nectar surface distance is longer than the groove length, the bill tips would not further compress the basal portion of the tongue grooves, and the collapsed configuration required for elastic pumping will be lost. Based purely on tongue filling efficiency, it would seem that longer bills would generally be better suited to optimize nectar extraction (Montgomerie 1984). However, compared to relatively shorter beaks (when controlling for effects of body size and associated musculature scaling), longer bills would be more difficult to wield (e.g., Montgomerie 1984; Temeles 1996; Collins 2008) due to increased torque especially when feeding from horizontal flowers. Longer-billed hummingbirds would take more time to position their bill tips adequately, and would make more insertion errors when feeding in narrow flowers, as compared to shorter-billed hummingbirds (see Montgomerie 1984; Temeles 1996). Insertion errors increase handling time (*i.e.*, the total time per floral visit), and would thus diminish net energy gain (Temeles 1996). Longer bills may also require longer nectar transport times after nectar off-loading near the bill tip (see Rico-Guevara 2014). Finally, longer bills are generally larger overall, and their increased bill and tongue tips thicknesses may impose additional problems at the moment of traversing very narrow corolla openings and/or nectar chamber access points. On the flipside, longer-billed hummingbirds also have beaks and tongues with a greater volumetric capacity for extracting nectar, perhaps thus requiring fewer licks to collect a given amount of nectar.

However, the example discussed above only applies to bill length, which is only one axis of variation of bill-corolla coevolution, and further evaluation of the biomechanical influence of the morphological variation in features such as bill curvature, tapering, and bill tip modifications (e.g., Rico-Guevara and Rubega 2017), is necessary. A productive starting point would be feeding efficiency studies based on experiments in which artificial flowers are used to control for different aspects of shape (*e.g.*, corolla length, curvature, and width; Temeles 1996; Collins 2008; Temeles et al. 2009; Rico-Guevara 2014). However, in order to disentangle these factors involved in nectar extraction efficiency, high-resolution and comprehensive morphological, biomechanical, and behavioral measurements on hummingbirds feeding on wild flowers will be essential (e.g., employing high-speed cameras; Rico-Guevara and Mickley 2017). It is also plausible that some birds escape the constraints of bill-corolla matching through nectar robbing and interference competition. Considering nectar extraction efficiency is crucial here, because net energy gain stands at the crux of various assumptions underlying explanations of ecological patterns and coevolutionary models for nectar-feeding birds.

## Recent advances in our understanding of selective forces acting on bill morphology

Hummingbirds have been proposed as one of the few examples of ecological causation of sexual dimorphism in nature (Temeles et al. 2000, 2010). In particular, it has been suggested that sexual differences in foraging behavior drive dimorphism in hummingbird beak shape (Temeles et al., 2000; 2005; Berns and Adams 2010, 2013), and as such the bill-corolla matching seen among hummingbird species has been inferred to pertain to different sexes of the same species as they partition floral resources (see Temeles et al. 2009; 2010). However, multiple selective forces may operate synergistically in the evolution of this sexually dimorphic trait, although the hypothesis of ecological causation has been widely promulgated (see Temeles and Roberts 1993; Bleiweiss 1999; Temeles et al. 2000, 2010). In contrast, we hypothesize that sexual dimorphism in hummingbird bill morphology may also reflect biomechanical tradeoffs between nectar feeding performance and physical competition among males competing for territories and mates (Rico-Guevara and Araya-Salas 2015; Hurme and Rico-Guevara 2016). Specifically, we propose that bill sexual dimorphism is related to fighting, and that structures like backward serrations, hooks, and daggers (e.g., Rico-Guevara and Rubega 2017), meet the conditions to be classified as intrasexually selected weapons (*sensu*Rico-Guevara and Hurme 2018). We have not found support for alternative hypotheses explaining the existence of these structures. Some of these hypotheses include that tomial serrations are adaptations for plumage preening, and that bill hooks and serrations are adaptations for flower-piercing (Ornelas 1994). Nonetheless, there is no evidence that there are differences in preening between males of serrated and unserrated species, in fact hummingbirds in general use their legs more than their bills for preening compared to other birds (Clayton and Cotgreave 1994). Furthermore, there is no relationship between the presence of dimorphic tomial serrations and bright vs. dull plumages, and/or plumage sexual dimorphism (see species surveyed by Rico-Guevara and Rubega 2017). Regarding the flower-piercing hypothesis (Ornelas 1994), this idea was proposed before it was taken into consideration that the expression of the tomial serrations is sexually dimorphic (e.g., Rico-Guevara 2014). Consequently, one would expect differences in nectar robbing between the sexes. However, males of serrated species have not been reported to pierce more than females, and the reports of nectar robbing are not skewed toward hummingbird species with serrated tomia (Robert Colwell personal communication). Additionally, hummingbird species that are considered specialized piercers (e.g., Wedge-billed Hummingbird [*Schistes geoffroyi*], Purple-crowned fairy [*Heliothryx barroti*]) do not present these sexually dimorphic serrations (Rico-Guevara and Rubega 2017). A thorough discussion of these and other alternative hypotheses for the evolution of serrated tomia in hummingbirds will be provided elsewhere (Gary Stiles personal communication).

One challenge of morphological and biomechanical studies is to quantify potential cost-benefit relationships among traits arising from intrasexual selection that are also subject to natural selective pressures. In this case in particular, our challenge will be to test if supplemental use of the hummingbird bill as a weapon reduces its performance as a tool for nectar feeding (Hurme and Rico-Guevara 2016), and to examine directly how mechanical trade-offs in different bill tip configurations shape hummingbird ecology, relative to benefits in either feeding or fighting.

**Fig. 4:**
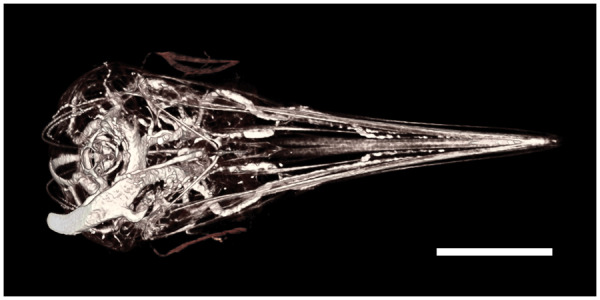
Hummingbird head vasculature. Ventral view of a Micro CT scan of a juvenile Broad-tailed Hummingbird (*Selasphorus platycercus*), courtesy of M. Scott Echols, DVM, Dipl. ABVP; scale bar = 5 mm. This specimen was perfused with BriteVu^®^ (by Scarlet Imaging), a contrast agent that allows visualization of the vasculature down to the capillary level. A rotation movie of this three-dimensional rendering can be accessed in the Supplementary Data (Video S1).

**Fig. 5:**
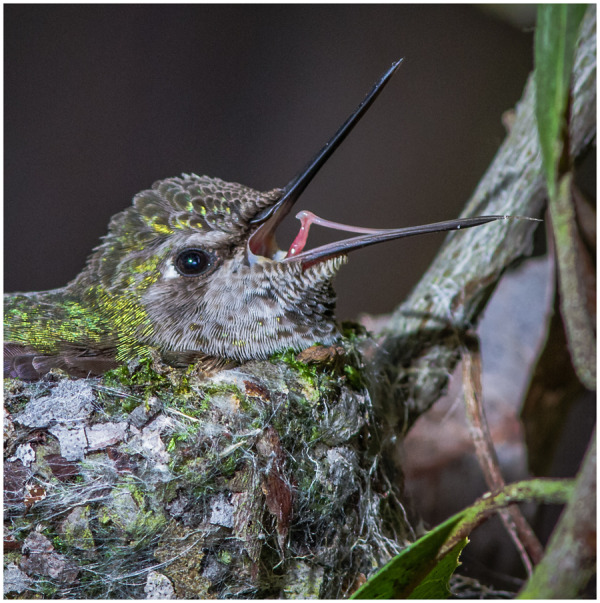
Hummingbird tongue lifting. Photograph of a female Anna’s Hummingbird (*Calypte anna*) at her nest, courtesy of Kim Michaels, www.KimMichaels.com. Notice that when the tongue is raised by the bird, it is only the base that it is actually lifted. The rest of the tongue lies inert on top of the mandible; hummingbirds do not have intrinsic muscles or skeletal support beyond the tongue base (e.g., Rico-Guevara 2017).

**Fig. 6:**
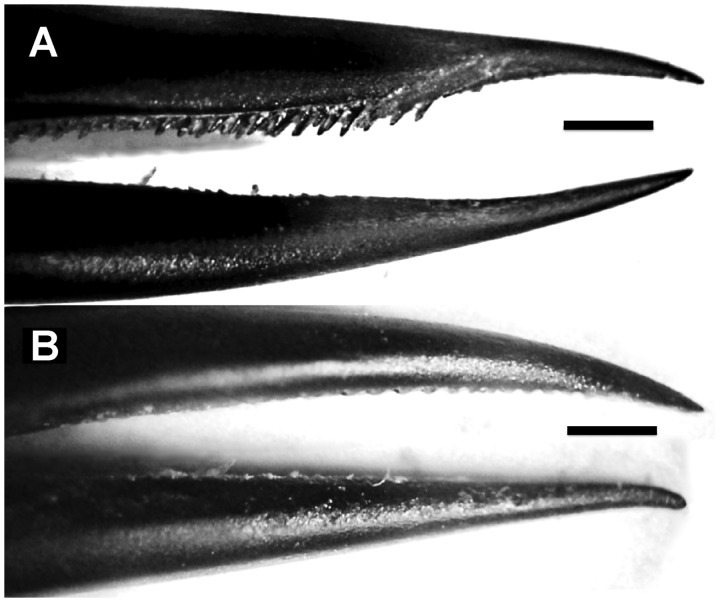
Sexual dimorphism in hummingbird bill tips. Photographs of Sparkling Violetear Hummingbird (*Colibri coruscans*) specimens. Scale bars = 1 mm. (A) An adult male showing dagger-like tips (i.e., pointy and conical; see Rico-Guevara and Araya Salas 2015) and backward-facing serrations. (B) An adult female showing the more typical hummingbird bill tip configuration (e.g., Rico-Guevara and Rubega 2017), with flexible and blunt bill tips (internally concave), and lacking backward serrations.

In particular, morphological changes to the hummingbird beak to facilitate use as a weapon are likely to impose trade-offs on nectar drinking efficiency:

1. When loaded axially, elongated structures are mechanically more resistant to buckling if they are straight (Kuo and Yang 1991; Dahlberg 2004). Bending is disadvantageous for a stabbing weapon, as less force is applied at the tip and subsequently less damage can be done to an opponent. In hummingbirds, straighter bills transmit more force without bending, and pointier bills transform that force into perforation capacity (Rico-Guevara and Araya-Salas 2015). In addition, bills used as weapons are expected to be reinforced to resist bending forces at the base (e.g., greater bill heights). However, such bill thickening might impair the ability of males from reaching deeper insider elongate flowers. In addition, stabbing collisions could transmit harmful forces to the skull, which then would be expected also to be reinforced depending on the strains produced by such forces. Predictions about force transmission and puncture capacity can be tested through three-dimensional finite element modeling, using microCT scans of hummingbird heads (such as those presented in Rico-Guevara 2017). In addition, the influence of varying morphology (e.g., bill thickness, curvature) on nectar extraction can be tested through multiple intra and interspecific comparisons controlling for confounding factors (e.g., overall size, bill length).
2. Hummingbird bills are very flexible to allow for both mandibular (Yanega and Rubega 2004; Yanega 2007; Smith et al. 2011) and maxillary bending (Rico-Guevara and Rubega 2011; Zusi 2013; Rico-Guevara 2014). Under the axial loads likely to be exerted when the bill is used as a stabbing weapon, however, such structural flexibility is likely to cause buckling and even failure. Consequently, we expect bills of males to present higher flexural rigidity when compared to those of females and juveniles, yet to retain enough flexibility to avoid breaking from impacts, and to continue to function for nectar extraction. Maxillary bending improves nectar drinking by allowing for opening of the bill tips while the middle portion of the beak remains tightly closed, which in turn enhances intraoral transport (Rico-Guevara 2014). Therefore, we predict that increased flexural rigidity (*i.e.*, stiffer beaks) will improve stabbing performance by diminishing bill bending, which concomitantly will reduce their ability to open the bill tip while keeping the rest of the beak shut, negatively influencing nectar-drinking efficiency. Predictions about flexural rigidity can be tested with microCT scans and close-up high-speed videos of the bill bending magnitude during feeding (e.g., Rico-Guevara 2014), along with estimations of the effect on feeding extraction efficiency.
3. Prior work has shown that, in order to extrude a nectar load from the tongue in an efficient manner, the internally concave bill tips squeeze the tongue and release the nectar inside the beak, preparing the grooves to collect as much nectar as possible on the following lick (see Rico-Guevara 2017; Rico-Guevara and Rubega 2017). Critically, it is important to appreciate that major regions of the hummingbird feeding apparatus are not under intrinsic muscular control as they interact with the flower and nectar (e.g., Figs. 1 and 2). The distal portions of the bill are composed mostly of keratin fitting tightly to the underlying bone (Rico-Guevara 2017), and vascularization extends only into the bill but not through the tongue (Fig. 4, Supplementary Video S1). The tongue itself is supported by unfused paraglossals (a rare condition in birds) for only one millimeter at the base (Supplementary Video S1). The rest of the tongue, and particularly its distal half, is entirely composed of keratin (Rico-Guevara 2017). Thus, hummingbirds only have control over the movement of the tongue base (Fig. 5), whilst the rest of the tongue will just follow the movements at the base and would need to be guided by the bill tips. It is only the interaction among the inert but dynamic portions of the bill and tongue tips that determines nectar extrusion and groove preparation. The concave bill tips fold and flatten the tongue grooves upon extrusion, allowing for a highly efficient elastic pumping mechanism (Rico-Guevara et al. 2015). Thus, sexually dimorphic and conical bill tips (Fig. 6) are predicted to improve piercing performance during stabbing, but should also reduce intraoral nectar offloading. The prediction that nectar unloading should be diminished in males of species with more conical bill tips can be tested by measuring the degree of tongue-flattening during extrusion (e.g., Rico-Guevara et al. 2015). Individuals with more conical bills should, on average, reduce the thickness of the tongue to a lesser degree than do individuals with less conical bills.
4. Similarly, flexible cutting edges on the bill (i.e., the tomia), with forward-facing serrations stay in close contact with the tongue surface upon extrusion, cleaning the tongue and maintaining nectar inside the bill (Rico-Guevara and Rubega 2017). However, tomial serrations in males of some species are stiffer and are directed backwards (e.g. Fig. 6;Hurme and Rico-Guevara 2016), which may be advantageous for biting and feather plucking from opponents during chases, but which may also disadvantage the process of nectar drinking given that modifications to stiffen the tomia for fighting will concomitantly diminish the bill tip’s squeezing capabilities (Rico-Guevara and Rubega 2017). Predictions about the influence of tomial flexibility on nectar extrusion can be tested by taking advantage of the existence of variation among adult males on the stiffness of their serrations, comparing feeding efficiency of different individuals, controlling for confounding factors such as bill length.
5. For most hummingbirds, the tongue and flexible tomia constantly are in contact; as a result, the tongue suffers visible wear in the form of fringed edges along distal portions of the grooves (Rico-Guevara 2014). The tongue grooves grow from their base continuously and their distal portions are kept at a constant length due to wear by the bill tips during drinking (Weymouth et al. 1964; Rico-Guevara 2017). When the tomia bear backward-facing, stiff serrations (e.g., as with the male in Fig. 6), we expect the tongue to undergo greater wear. To confirm this expectation, close-up videos of the bill-tongue interaction in such species are needed to determine the amount of contact between serrations and tongue walls, which would vary with tomial orientation during feeding. We have observed darker tongues in some species with “weaponized” bills during anatomical surveys of hummingbird taxa (described in Rico-Guevara and Rubega 2011 and Rico-Guevara 2014). We thus hypothesize that dark coloration of the tongue is the result of higher concentrations of melanin to help withstand abrasion (Barrowclough and Sibley 1980; Bonser 1995) during persistent wear against the stiff and sharp tomia. Greater melanization of the tongue should also reduce its flexibility, and thus alter its ability to store potential energy as a nectar trap (Rico-Guevara and Rubega 2011) and as an elastic micropump (Rico-Guevara et al. 2015).

In sum, adult male hummingbirds with “weaponized” bills should either exhibit reduced nectar feeding performance (as compared to conspecific females and juveniles), or they may exhibit altered biomechanical performance to compensate for changes in bill shape and for their relatively stiffer bill tips and tongue tissue. The prediction that more weaponized bills impose more tongue wear, and that tongues are reinforced to withstand it, could be tested by examining the degree of melanization in the tongues of weaponized vs. unweaponized species. The prediction that nectar extraction efficiency will be reduced could be tested with experiments evaluating nectar collection rates among several cases of contrasting armed vs. unarmed species. Furthermore, it is important to highlight that since we are basing our assumptions on basic physical principles, we expect a continuum of performance, based on the degree of weaponization, not necessarily just sexual or interspecific differences.

We have filmed hummingbird fights during which territorial males stab, bite, and pluck feathers from intruders with their bills, and we have documented bill tip modifications in males for some territorial species (Fig. 6; Rico-Guevara 2014; Rico-Guevara and Araya-Salas 2015; Hurme and Rico-Guevara 2016) such that these bills can be considered to be intrasexually selected weapons (Rico-Guevara and Araya-Salas 2015; Rico-Guevara and Hurme 2018). Definitive proof of the use of hummingbird beaks as stabbing weapons would come from detailed biomechanical analyses of physical contact in the form of high-speed videos of direct stabs, and of surveys on wounds after these fights. Nonetheless, there are many structures used as weapons in nature (review in Rico-Guevara and Hurme 2018) that do not leave visible wounds on the combatants; in hummingbirds, plucked or damaged feathers could be the most common result from physical confrontations, rather than open wounds (which could nevertheless occur). Fighting in hummingbirds is an emerging area of study, and we are far from establishing if the confrontations would commonly lead to visible wounds. Based on our field observations, there seem to exist a variety of visual (e.g., puffing facial feathers, pausing their hovering momentarily to keep their wings open) and acoustic (e.g., aggressive calls) displays to avoid conflict escalation, and when there are physical fights, they quickly turn into chases in which the plumage is reached and damaged before the skin would (A. Rico-Guevara, Personal observation). Additionally, open wounds or scars may well go undetected, unless researchers are particularly looking for them, because the feathers would readily cover them. Finally, a severely wounded bird would likely be even more difficult to detect, since researchers would be unlikely to encounter or trap a bird too wounded to fly, or bleeding too profusely to have the energy to move.

Across all hummingbird clades, there are seven independent instances of evolution of bill tip modifications that we propose to be weapons, namely daggers, hooks, and backward serrations (Rico-Guevara 2014; Hurme and Rico-Guevara 2016). These are likely intrasexually selected weapons, as only the males have bills either tipped with daggers (Rico-Guevara and Araya-Salas 2015) or possessing hooks and/or backward serrations (Rico-Guevara 2014; Rico-Guevara and Rubega 2017). None of these sexual differences (e.g., Fig. 6) would be expected under the hypothesis of ecological causation; only straighter bills for males have been proposed to derive from intersexual resource partitioning (see Temeles et al. 2010). Ecological causation may not be, however, the only mechanism by which sexual dimorphism in bill morphology is achieved. Sexual selection in the form of male-male competition may also constrain bill curvature because straighter bills can be better weapons. It is also possible that small differences in advantages to hummingbird-pollinated plants with curved corollas are enhanced via niche partitioning, reinforcing benefits to bill curvature in females while subjecting males to a performance trade-off. In most of the independent occurrences of sexual dimorphism in bill curvature, male hummingbirds have straighter beaks relative to females (Temeles et al. 2010), which would not be expected under scenarios that were purely driven by ecological (*i.e.*, non-sexually selected) factors.

“Weaponized” or armed males may thus face trade-offs in nectar extraction efficiencies, but they may be also favored by enhanced odds of winning when engaged in both intra- (Rico-Guevara and Araya-Salas 2015) and interspecific combat (e.g., Hurme and Rico-Guevara 2016). As already noted, different hummingbird species, and males and females of the same species, use different strategies to access nectar, and both bill size and shape sexual dimorphism seem to have occurred early in hummingbird diversification (Berns and Adams 2013). All hummingbirds feed on floral nectar, such that each species of hummingbird competes to some degree against all other local hummingbird taxa. As a result of similarities in morphology, habitat preferences, and foraging behavior, we might expect greater competition between two members of the same clade than between two hummingbird species from different clades. Competition would be further increased between two species with the same foraging role (*sensu*Feinsinger and Colwell 1978, who suggested that within any one assemblage there should be more competition between species classified in the same role than between species in different roles). One of the most common hummingbird foraging strategies is to exclude competitors from territories through aggressive interactions facilitated by various morphological and behavioral traits (Stiles 1981; González-Gómez et al. 2014; Martin and Ghalambor 2014; Lanna et al. 2017). Moreover, a hummingbird’s ability to defend a territory indirectly results in enhanced fitness through increased physiological capacity (Márquez-Luna et al. 2014) and/or female choice on territory quality. Therefore, we suggest that future research should test the hypothesis that males of weaponized species (see Fig. 6), ceteris paribus, will rank higher in dominance hierarchies (e.g., Justino et al. 2012) than non-armed species, when controlling for body size and other factors that could influence fight outcomes. In such assessment, holistic appraisal of the morphological adaptations (beyond bill shape) for fighting vs. energetic optimality strategies is warranted. For instance, we would expect that dominant morphs (e.g., adult males vs. juveniles and females), would exhibit reduced aerodynamic efficiency relative to subordinate ones, and that this would select for different optima in associated traits (e.g., wing area, wing aspect ratio, and muscle capacity). As an example of one of these traits, relatively larger wings can increase fighting performance (e.g., by increasing body accelerations; e.g., Dakin et al. 2018), while relatively smaller wings would be more efficient for increased displacement times (e.g., subordinate morphs travelling longer among food patches).

## Concluding remarks

Hummingbirds represent a model clade with which to study interactions between the physics of foraging and feeding with ecological context, and with the evolution of novel morphologies and diverse behaviors. For instance, hummingbirds are one of the few taxa in which intrasexually selected weapons are modifications of the feeding apparatus with opposing biomechanical pressures (*i.e.*, the trade-offs described above). Most sex-specific weapons that are direct modifications of feeding apparati with retained feeding functionality have transitioned to enhance their original biomechanical function, for instance cutting in herbivores and piercing in faunivores (Rico-Guevara and Hurme 2018). This exceptional situation in hummingbirds offers an excellent opportunity to study the trade-offs between natural and sexual selection, and between exploitative and interference competition. We have proposed here a series of integrative approaches that can yield new quantitative data previously unattainable for this system. These studies can yield a better understanding of hummingbird-plant coevolutionary relationships, beginning with a comprehensive understanding of the biophysics of nectar collection, and extending to large-scale ecological patterns (including exploitative and interference competition) and evolutionary dynamics. The functional framework proposed here provides testable predictions about measurable trade-offs in the evolution of hummingbird bills. For instance, “weaponized” bill features derive from their use in male-male competition, whereas sexual dimorphism in bill characteristics underpins resource partitioning between the sexes. Variable interpretations of the costs of intrasexually selected weapons (see Kotiaho 2001; McCullough and Emlen 2013) stem from difficulties in the direct measurement of energetic impacts; since the biomechanics of hummingbird feeding are now understood in detail, and because hummingbird weapons are a direct modification of the feeding apparatus, this system is well poised to directly measure such costs. Both theoretical and experimental tools can assist us in evaluating the implications of our highlighted paradigm shifts (*i.e.*, mechanics of nectar collection, evolution of bill sexual dimorphism) for reassessment of the current framework of hummingbird-plant networks and coevolution. More generally, hummingbird nectarivory and associated floral specialization are highly derived and paradigmatic examples of vertebrate foraging behavior, for which we can now provide mechanistic analyses relative to the costs of both feeding and aerial movement.

## Supplementary Material

Supplementary Data

## References

[oby006-B2] Abrahamczyk S, Renner SS. 2015 The temporal build-up of hummingbird/plant mutualisms in North America and temperate South America. BMC Evol Biol 15:104. 26058608 10.1186/s12862-015-0388-z PMC4460853

[oby006-B3] Abrahamczyk S, Souto-Vilarós D, Renner SS. 2014 Escape from extreme specialization: passionflowers, bats and the sword-billed hummingbird. Proc R Soc B Biol Sci 281:20140888. 10.1098/rspb.2014.0888 PMC4213610 25274372

[oby006-B5] Araya-Salas M, Gonzalez-Gómez P, Wojczulanis-Jakubas K, López V, Wright TF. 2018 Spatial memory is as important as weapon and body size for territorial ownership in a lekking hummingbird. Scientific Rep 8:1–11. 10.1038/s41598-018-20441-x PMC5792557 29386557

[oby006-B6] Baker HG. 1975 Sugar concentrations in nectars from hummingbird flowers. Biotropica 7:37–41.

[oby006-B7] Baldwin MW, Toda Y, Nakagita T, O'Connell MJ, Klasing KC, Misaka T, Liberles SD. 2014 Evolution of sweet taste perception in hummingbirds by transformation of the ancestral umami receptor. Science 345:929–33. 25146290 10.1126/science.1255097 PMC4302410

[oby006-B8] Barrowclough GF, Sibley FC. 1980 Feather pigmentation and abrasion: test of a hypothesis. The Auk 97:881–83.

[oby006-B9] Bateson M, Healy SD, Hurly TA. 2003 Context-dependent foraging decisions in rufous hummingbirds. Proc R Soc B Biol Sci 270:1271–76. 10.1098/rspb.2003.2365 PMC1691372 12816640

[oby006-B10] Baum KA, Grant WE. 2001 Hummingbird foraging behavior in different patch types: simulation of alternative strategies. Ecol Model 137:201–9.

[oby006-B12] Berns CM, Adams DC. 2010 Bill shape and sexual shape dimorphism between two species of temperate hummingbirds: black-chinned hummingbird (*Archilochus alexandri*) and Ruby-Throated hummingbird (*A. colubris*). The Auk 127:626–35.

[oby006-B13] Berns CM, Adams DC. 2013 Becoming different but staying alike: patterns of sexual size and shape dimorphism in bills of hummingbirds. J Evol Biol 40:246–60.

[oby006-B15] Bleiweiss R. 1999 Joint effects of feeding and breeding behaviour on trophic dimorphism in hummingbirds. Proc R Soc B Biol Sci 266:2491–7. 10.1098/rspb.1999.0951 PMC1690480 10693820

[oby006-B16] Blem CR, Blem LB, Cosgrove CC. 1997 Field studies of rufous hummingbird sucrose preference: does source height affect test results? J Field Ornithol 68:245–52.

[oby006-B17] Blem CR, Blem LB, Felix J, van Gelder J. 2000 Rufous hummingbird sucrose preference: precision of selection varies with concentration. Condor 102:235–8.

[oby006-B18] Bonser RH. 1995 Melanin and the abrasion resistance of feathers. Condor 97:590.

[oby006-B19] Bowman RI. 1961 Morphological differentiation and adaptation in the Galapagos Finches. Univ California Pub Zool 58:1–302.

[oby006-B20] Calder WA. 1979 On the temperature-dependency of optimal nectar concentration for birds. J Theoret Biol 78:185–96. 491711 10.1016/0022-5193(79)90263-7

[oby006-B21] Chai P, Dudley R. 1999 Maximum flight performance of hummingbirds: capacities, constraints, and trade-offs. The Am Natural 153:398–411.

[oby006-B23] Clayton DH, Cotgreave P. 1994 Relationship of bill morphology to grooming behaviour in birds. Anim Behav 47:195–201.

[oby006-B24] Collins BG. 2008 Nectar intake and foraging efficiency: responses of honeyeaters and hummingbirds to variations in floral environments. The Auk 125:574–87.

[oby006-B25] Cooney CR, Bright JA, Capp EJR, Chira AM, Hughes EC, Moody CJA, Nouri LO, Varley ZK, Thomas GH. 2017 Mega-evolutionary dynamics of the adaptive radiation of birds. Nature 542:344–7. 28146475 10.1038/nature21074 PMC5321581

[oby006-B26] Dahlberg T. 2004 Procedure to calculate deflections of curved beams. Int J Eng Edu 20:503–13.

[oby006-B27] Dakin R, Segre PS, Straw AD, Altshuler DL. 2018 Morphology, muscle capacity, skill, and maneuvering ability in hummingbirds. Science 359:653–7. 29439237 10.1126/science.aao7104

[oby006-B28] Darwin C. 1841 The zoology of the voyage of H.M.S. Beagle: Part III Birds. London: Smith, Elder and Co p. 156.

[oby006-B30] DeBenedictis PA, Gill FB, Hainsworth F R, Pyke GH, Wolf LL. 1978 Optimal meal size in hummingbirds. The Am Natural 112:301–16.

[oby006-B32] Ewald PW, Williams WA. 1982 Function of the bill and tongue in nectar uptake by hummingbirds. The Auk 99:573–76.

[oby006-B33] Feinsinger P, Colwell RK. 1978 Community organization among Neotropical nectar-feeding birds. Am Zool 18:779–95.

[oby006-B35] Fleming PA, Hartman Bakken B, Lotz CN, Nicolson SW. 2004 Concentration and temperature effects on sugar intake and preferences in a sunbird and a hummingbird. Funct Ecol 18:223–32.

[oby006-B37] Forister ML, Dyer LA, Singer MS, Stireman JO, Lill JT. 2012 Revisiting the evolution of ecological specialization, with emphasis on insect–plant interactions. Ecology 93:981–91. 22764485 10.1890/11-0650.1

[oby006-B38] Futuyma DJ. 2009 Evolution. Massachusetts: Sinauer Associates p. 614.

[oby006-B39] Futuyma DJ, Moreno G. 1988 The evolution of ecological specialization. Ann Rev Ecol Syst 19:207–33.

[oby006-B41] Gass CL, Roberts WM. 1992 The problem of temporal scale in optimization: Three contrasting views of hummingbird visits to flowers. Am Nat 140:829–53. 19426045 10.1086/285443

[oby006-B43] González-Gómez PL et al 2014 Cognitive ecology in hummingbirds: the role of sexual dimorphism and its anatomical correlates on memory. PloS One 9:e90165. 24599049 10.1371/journal.pone.0090165 PMC3943908

[oby006-B45] Grant V, Temeles EJ. 1992 Foraging ability of rufous hummingbirds on hummingbird flowers and hawkmoth flowers. Proc Natl Acad Sci USA 89:9400–4. 11607331 10.1073/pnas.89.20.9400 PMC50139

[oby006-B46] Gregory TR, Andrews CB, McGuire JA, Witt CC. 2009 The smallest avian genomes are found in hummingbirds. Proc R Soc B Biol Sci 276:3753–7. 10.1098/rspb.2009.1004 PMC2817281 19656792

[oby006-B48] Hainsworth FR. 1973 On the tongue of a hummingbird: Its role in the rate and energetics of feeding. Comp Biochem Physiol A Physiol 46:65–78. 10.1016/0300-9629(73)90559-8 4147805

[oby006-B51] Hainsworth FR, Wolf LL. 1976 Nectar characteristics and food selection by hummingbirds. Oecologia 25:101–13. 28308993 10.1007/BF00368847

[oby006-B54] Hedrick TL, Cheng B, Deng X. 2009 Wingbeat time and the scaling of passive rotational damping in flapping flight. Science 324:252–5. 19359586 10.1126/science.1168431

[oby006-B56] Heyneman AJ. 1983 *.* Optimal sugar concentrations of floral nectars—dependence on sugar intake efficiency and foraging costs. Oecologia 60:198–213. 28310487 10.1007/BF00379522

[oby006-B58] Hixon MA, Carpenter FL. 1988 *.* Distinguishing energy maximizers from time minimizers: a comparative study of two hummingbird species. Am Zool 28:913–25.

[oby006-B60] Houston AI, McNamara JM. 2014 Foraging currencies, metabolism and behavioural routines. J Anim Ecol 83:30–40. 23730810 10.1111/1365-2656.12096

[oby006-B62] Hurme K, Rico-Guevara A. 2016 Little hungry warriors: examining trade-offs between fighting and feeding in hummingbirds. Accessed May 15, 2016 at experiment.com/fightinghummingbirds DOI: 10.18258/6810.

[oby006-B63] Janzen DH. 1980 When is it coevolution. Evolution 34:611–2. 28568694 10.1111/j.1558-5646.1980.tb04849.x

[oby006-B64] Justino DG, Maruyama PK, Oliveira PE. 2012 Floral resource availability and hummingbird territorial behaviour on a Neotropical savanna shrub. J Ornithol 153:189–97.

[oby006-B65] Kim W, Gilet T, Bush JW. 2011 Optimal concentrations in nectar feeding. Proc Natl Acad Sci USA 108:16618–21. 21949358 10.1073/pnas.1108642108 PMC3189050

[oby006-B66] Kim W, Peaudecerf F, Baldwin MW, Bush JW. 2012 The hummingbird's tongue: a self-assembling capillary syphon. Proc R Soc B Biol Sci 279:4990–6. 10.1098/rspb.2012.1837 PMC3497234 23075839

[oby006-B67] Kingsolver JG, Daniel T. L. 1983 Mechanical determinants of nectar feeding strategy in hummingbirds: energetics, tongue morphology, and licking behavior. Oecologia 60:214–26. 28310488 10.1007/BF00379523

[oby006-B68] Kotiaho SJ. 2001 Costs of sexual traits: a mismatch between theoretical considerations and empirical evidence. Biol Rev Cambridge Philos Soc 76:365–76. 11569789 10.1017/s1464793101005711

[oby006-B69] Krauss SL, Phillips RD, Karron JD, Johnson SD, Roberts DG, Hopper SD. 2017 Novel Consequences of Bird Pollination for Plant Mating. Trends Plant Sci 5:395–410. 10.1016/j.tplants.2017.03.005 28412035

[oby006-B70] Kuo SR, Yang YB. 1991 New theory on buckling of curved beams. J Eng Mechanics 117:1698–717.

[oby006-B71] Lanna LL, Azevedo CSD, Claudino RM, Oliveira R, Antonini Y. 2017 Feeding behavior by hummingbirds (Aves: Trochilidae) in artificial food patches in an Atlantic Forest remnant in southeastern Brazil. Zoologia (Curitiba) 34:e13228.

[oby006-B72] Lara C, Ornelas J. 2001 Preferential nectar robbing of flowers with long corollas: experimental studies of two hummingbird species visiting three plant species. Oecologia 128:263–73. 28547475 10.1007/s004420100640

[oby006-B73] Lasiewski RC. 1963 Oxygen consumption of torpid, resting, active, and flying hummingbirds. Physiol Zool 36:122–40.

[oby006-B74] Lucas FA. 1891 On the structure of the tongue in humming birds. Proc Natl Acad Sci USA 14:169–72.

[oby006-B75] Lunau K. 2004 Adaptive radiation and coevolution-pollination biology case studies. Organ Diversity Evol 4:207–24.

[oby006-B78] Maglianesi MA, Bluthgen N, Bohning-Gaese K, Schleuning M. 2014 Morphological traits determine specialization and resource use in plant-hummingbird networks in the neotropics. Ecology 95:3325–34.

[oby006-B80] Márquez-Luna U, Lara C, Ortiz-Pulido R. 2014 La conducta territorial del Zafiro oreja blanca Hylocharis leucotis) es afectada por la disponibilidad de energía. Ornitologia Neotropical 25:433–43.

[oby006-B82] Martin PR, Ghalambor CK. 2014 When David beats Goliath: the advantage of large size in interspecific aggressive contests declines over evolutionary time. PloS One 9:e108741. 25250781 10.1371/journal.pone.0108741 PMC4177554

[oby006-B83] Martin WCL. 1833 The naturalist’s library: a general history of humming-birds or the Trochilidae. London: H. G. Bohn.

[oby006-B84] Martínez del Río C. 1990 . Sugar preferences in hummingbirds: the influence of subtle chemical differences on food choice. Condor 92:1022–30.

[oby006-B85] McCullough EL, Emlen DJ. 2013 Evaluating the costs of a sexually selected weapon: big horns at a small price. Anim Behav 86:977–85.

[oby006-B90] Montgomerie RD. 1984 Nectar extraction by hummingbirds: Response to different floral characters. Oecologia 63:229–36. 28311018 10.1007/BF00379882

[oby006-B96] Nicolson SW. 2002 Pollination by passerine birds: why are the nectars so dilute? Comp Biochem Physiol B Biochem Mol Biol 131:645–52. 11923080 10.1016/s1096-4959(02)00014-3

[oby006-B97] Nicolson SW. 2007 Nectar consumers In: Nepi M, Pacini E, editors. Nectaries and nectar. Netherlands: Springer p. 289–342.

[oby006-B98] Nicolson SW, Fleming PA. 2003 Nectar as food for birds: the physiological consequences of drinking dilute sugar solutions. Plant Syst Evol 238:139–53.

[oby006-B99] Nicolson SW, Fleming PA. 2014 Drinking problems on a ‘simple’ diet: physiological convergence in nectar-feeding birds. J Exp Biol 217:1015–23. 24671960 10.1242/jeb.054387

[oby006-B100] Nicolson SW, Thornburg RW. 2007 Nectar chemistry In: Nepi M, Pacini E, editors. Nectaries and nectar. Netherlands: Springer p. 215–64.

[oby006-B101] Olsen AM. 2017 Feeding ecology is the primary driver of beak shape diversification in waterfowl. Funct Ecol 2017:1–11.

[oby006-B102] Ornelas JF. 1994 Serrate tomia: an adaptation for nectar robbing in hummingbirds?. The Auk 111:703–10.

[oby006-B106] Proctor M, Yeo P, Lack A. 1996 The natural history of pollination. Portland (OR): Timber Press p. 479.

[oby006-B112] Pyke GH, Waser NM. 1981 The production of dilute nectars by hummingbird and honeyeater flowers. Biotropica 13:260–70.

[oby006-B117] Rico-Guevara A. 2014 Morphology and function of the drinking apparatus in hummingbirds [dissertation]. Storrs (CT): University of Connecticut. p. 241 Available from: http://digitalcommons.uconn.edu/dissertations/490.

[oby006-B118] Rico-Guevara A. 2017 Relating form to function in the hummingbird feeding apparatus. PeerJ 5:e3449. 10.7717/peerj.3449. 28607842 PMC5466813

[oby006-B119] Rico-Guevara A, Hurme K. 2018 Intrasexually selected weapons. Biol Rev. 10.1111/brv.12436. 29924496

[oby006-B120] Rico-Guevara A, Araya-Salas M. 2015 Bills as daggers? A test for sexually dimorphic weapons in a lekking hummingbird. Behav Ecol 26:21–29.

[oby006-B121] Rico-Guevara A, Rubega MA. 2011 The hummingbird tongue is a fluid trap, not a capillary tube. Proc Natl Acad Sci USA 108:9356–60. 21536916 10.1073/pnas.1016944108 PMC3111265

[oby006-B122] Rico-Guevara A, Rubega MA. 2012 Hummingbird feeding mechanics: Comments on the capillarity model. Proc Natl Acad Sci USA 109:E867. 22460801 10.1073/pnas.1119750109 PMC3326478

[oby006-B123] Rico-Guevara A, Rubega MA. 2017 Functional morphology of hummingbird bill tips: their function as tongue wringers. Zoology 123:1–10. 10.1016/j.zool.2017.06.001. 28760683

[oby006-B124] Rico-Guevara A, Fan TH, Rubega MA. 2015 Hummingbird tongues are elastic micropumps. Proc R Soc B Biol Sci 282:20151014. 10.1098/rspb.2015.1014 PMC4632618 26290074

[oby006-B125] Rico‐Guevara A, Mickley J. 2017 Bring your own camera to the trap: An inexpensive, versatile, and portable triggering system tested on wild hummingbirds. Ecol Evol 2017:1–7. 10.1002/ece3.3040 PMC5496556 28690789

[oby006-B126] Roberts WM. 1995 Hummingbird licking behaviour and the energetics of nectar feeding. The Auk 112:456–63.

[oby006-B127] Roberts WM. 1996 Hummingbirds’ nectar concentration preferences at low volume: The importance of time scale. Anim Behav 52:361–70.

[oby006-B128] Royama T. 1970 Factors governing the hunting behaviour and selection of food by the great tit (Parus major L.). J Anim Ecol 39:619–68.

[oby006-B129] Scharnke H. 1931 Contribution to the morphology and developmental evolution of the tongue of the Trochilidae, Meliphagidae and Picidae. J Ornithol 79:425–91.

[oby006-B130] Serrano-Serrano ML, Rolland J, Clark JL, Salamin N, Perret M. 2017 Hummingbird pollination and the diversification of angiosperms: an old and successful association in Gesneriaceae. Proc R Soc B Biol Sci 284:20162816. 10.1098/rspb.2016.2816 PMC5394660 28381621

[oby006-B131] Smith ML, Yanega GM, Ruina A. 2011 Elastic instability model of rapid beak closure in hummingbirds. J Theoret Biol 282:41–51. 21609721 10.1016/j.jtbi.2011.05.007

[oby006-B133] Stiles FG. 1976 Taste preferences, color preferences, and ﬂower choice in hummingbirds. Condor 78:10–26.

[oby006-B135] Stiles FG. 1981 Geographical aspects of bird-flower coevolution, with particular reference to Central America. Ann Missouri Botanical Garden 68:323–51.

[oby006-B137] Stiles FG, Freeman CE. 1993 Patterns in floral nectar characteristics of some bird-visited plant species from Costa Rica. Biotropica 25:191–205.

[oby006-B138] Stromberg MR, Johnsen PB. 1990 Hummingbird sweetness preference: taste or viscosity? Condor 92:606–12.

[oby006-B139] Suarez RK. 1992 Hummingbird flight: sustaining the highest mass-specific metabolic rates among vertebrates. Cell Mol Life Sci 48:565–70. 10.1007/BF01920240 1612136

[oby006-B140] Tamm S, Gass CL. 1986 Energy intake rates and nectar concentration preferences by hummingbirds. Oecologia 70:20–23. 28311283 10.1007/BF00377107

[oby006-B142] Temeles EJ. 1996 A new dimension to hummingbird-flower relationships. Oecologia 105:517–23. 28307145 10.1007/BF00330015

[oby006-B143] Temeles EJ, Koulouris CR, Sander SE, Kress WJ. 2009 Effect of flower shape and size on foraging performance and trade‐offs in a tropical hummingbird. Ecology 90:1147–61. 19537537 10.1890/08-0695.1

[oby006-B144] Temeles EJ, Roberts WM. 1993 Effect of sexual dimorphism in bill length on foraging behavior: an experimental analysis of hummingbirds. Oecologia 94:87–94. 28313864 10.1007/BF00317307

[oby006-B145] Temeles EJ, Goldman RS, Kudla AU. 2005 Foraging and territory economics of sexually dimorphic purple-throated caribs (*Eulampis jugularis*) on three *Heliconia* morphs. The Auk 122:187–204.

[oby006-B147] Temeles EJ, Miller JS, Rifkin JL. 2010 Evolution of sexual dimorphism in bill size and shape of hermit hummingbirds (Phaethornithinae): a role for ecological causation. Philos Trans R Soc B Biol Sci 365:1053–63. 10.1098/rstb.2009.0284 PMC2830232 20194168

[oby006-B148] Temeles EJ, Pan IL, Brennan JL, Horwitt JN. 2000 Evidence for ecological causation of sexual dimorphism in a hummingbird. Science 289:441–3. 10903203 10.1126/science.289.5478.441

[oby006-B149] Tobalske BW, Hedrick TL, Dial KP, Biewener AA. 2003 Comparative power curves in bird flight. Nature 421:363–6. 12540899 10.1038/nature01284

[oby006-B150] Tripp EA, McDade LA. 2013 Time-calibrated phylogenies of hummingbirds and hummingbird-pollinated plants reject a hypothesis of diffuse co-evolution. Aliso J Syst Evol Botany 31:89–103.

[oby006-B151] Tullock G. 1971 The coal tit as a careful shopper. Am Nat 105:77–80.

[oby006-B152] Van Riper W. 1958 Hummingbird feeding preferences. The Auk 75:100–1.

[oby006-B153] Vogel S. 2011 Surface tension helps a tongue grab liquid. Proc Natl Acad Sci USA 108:9321–2. 21610163 10.1073/pnas.1107208108 PMC3111306

[oby006-B154] Weinstein BG, Graham CH. 2017 Persistent bill and corolla matching despite shifting temporal resources in tropical hummingbird-plant interactions. Ecol Lett 20:326–35. 28150364 10.1111/ele.12730

[oby006-B155] Weis-Fogh T. 1972 Energetics of hovering flight in hummingbirds and in Drosophila. J Exp Biol 56:79–104.

[oby006-B156] Weymouth RD, Lasiewski RC, Berger AJ. 1964 The tongue apparatus in hummingbirds. Acta Anatomical 58:252–70. 10.1159/000142586 14262362

[oby006-B157] Wolf LL, Hainsworth FR. 1991 Hummingbird foraging patterns: visits to clumps of Ipomopsis aggregata inflorescences. Anim Behav 41:803–12.

[oby006-B158] Wolf LL, Hainsworth FR, Gill FB. 1975 Foraging efficiencies and time budgets in nectar-feeding birds. Ecology 56 117–28.

[oby006-B159] Wolf LL, Stiles FG, Hainsworth FR. 1972 Energetics of foraging: rate and efficiency of nectar extraction by hummingbirds. Science 176:1351–2. 17820919 10.1126/science.176.4041.1351

[oby006-B160] Wright NA, Gregory TR, Witt CC. 2014 Metabolic ‘engines’ of flight drive genome size reduction in birds. Proc R Soc B Biol Sci 281:1–9. 10.1098/rspb.2013.2780 PMC3924074 24478299

[oby006-B161] Yanega GM. 2007 A comparative study of the functional morphology and ecology of insectivory in hummingbirds [dissertation]. Storrs (CT): University of Connecticut. p. 295. Available from: http://digitalcommons.uconn.edu/dissertations/AAI3289529/.

[oby006-B162] Yanega GM, Rubega MA. 2004 Hummingbird jaw bends to aid insect capture. Nature 428:615. 15071586 10.1038/428615a

[oby006-B165] Zusi RL. 2013 Introduction to the skeleton of hummingbirds (Aves: Apodiformes, Trochilidae) in functional and phylogenetic contexts. Ornithol Monographs 77:1–94.

